# Cytogenotoxicity of food preservatives in mammalian cells: A systematic review

**DOI:** 10.1590/1678-4685-GMB-2025-0137

**Published:** 2025-12-15

**Authors:** Thiago Guedes Pinto, Daniel V. de Souza, Rogerio A. Dedivitis, Ana Claudia M. Renno, Daniel A. Ribeiro, Daisy M. F. Salvadori

**Affiliations:** 1 Universidade Federal de São Paulo (UNIFESP), Instituto de Saúde e Sociedade, Departamento de Biociências, Santos, SP, Brazil.; 2 Universidade de São Paulo (USP), Faculdade de Medicina, Laboratório de Cirurgia de Cabeça e Pescoço, São Paulo, SP, Brazil.; 3 Universidade Estadual Paulista (UNESP), Faculdade de Medicina, Departamento de Patologia, Botucatu, SP, Brazil.

**Keywords:** Genotoxicity, cytotoxicity, food preservatives, mammalian cells

## Abstract

This systematic review investigates the cytogenotoxicity of various food preservatives in mammalian cells, including sodium benzoate and potassium sorbate, through a comprehensive analysis of studies retrieved from PubMed, SCOPUS, and Web of Science. An orderly search conducted in March 2025 identified 19 relevant studies (from an initial 594), which employed assays, such as the micronucleus test and the comet assay to assess DNA damage, and MTT assay and polychromatic/normochromatic erythrocytes (PCE/NCE) ratio for evaluating cytotoxicity. Among these, 13 studies (68 %) reported genotoxic effects, with sodium benzoate being the most frequently associated with micronucleus formation and chromosomal abnormalities. Additionally, 12 studies (63 %) described cytotoxic effects, evidenced by decreased cell viability, altered proliferation indices, or nuclear alterations. As for the quality assessment, 18 studies (out of 19) were categorized as strong (n = 15) or moderate (n = 3) and, therefore, we consider our findings to be trustworthy. In summary, the consistent association between exposure to food preservatives and cytogenotoxic outcomes highlights the importance of monitoring such compounds and establishing clearer safety thresholds to protect human health. Certainly, these findings are important for clarifying the role of biomarkers related to cytogenotoxicity due to food preservative consumption in humans.

## Introduction

Food preservation, one of humanity’s oldest technologies, has evolved to incorporate the use of preservatives to extend freshness and shelf life while inhibiting oxidation, driven by the demands of a growing global population (Pandey et al., 2014). To this end, chemical preservatives, a category of food additives, are extensively utilized globally due to their apparent efficacy in preserving food (Pandey *et al.*, 2014). 

The underlying rationale that underscores the importance of using food preservatives is the fact that these food products are often distributed to regions far from their production sites (Llana-Ruiz-Cabello et al., 2016). Consequently, these foods become vulnerable to various reactions, such as microbial spoilage or oxidation processes, which can compromise the safety and organoleptic properties of perishable items (Llana-Ruiz-Cabello *et al.*, 2016). In this context, it is coherent to state that humans are consistently exposed to preservatives via the oral route, as these substances are known to inhibit or slow nutritional losses due to microbiological, enzymatic, or chemical changes in food (NTP, 2001; Zengin et al., 2011). 

Hence, although these substances are proven to extend the shelf life and maintain the quality of foods, research indicates that certain food preservatives may exhibit cytogenotoxicity in various test systems (Luca *et al.*, 1987; Mukherjee et al., 1988; Pandey et al., 2014). Therefore, it is essential to examine the effects of consumer exposure to these xenobiotics to assess their potential impact on human health (Croom, 2012). Examples of these potential carcinogenic chemical agents include nitrites, nitrates, butylated hydroxyanisole, potassium bromate, sorbate, among others (IARC, 2010). 

In terms of how these food preservatives reach individuals, it is known that they enter the body primarily through the ingestion of food via the mouth and, after ingestion; they are absorbed through the gastrointestinal tract and enter the bloodstream. After that, they are distributed throughout the body and reach the liver, the primary preservative metabolization site. Subsequently, these substances undergo metabolic transformations facilitated by various enzymatic actions, particularly those in the cytochrome P450 family. Lastly, following conjugation with other molecules, such substances are eliminated from the organism via urine and feces (Hodges and Minich, 2015; Gilani and Cassagnol, 2023).

Nevertheless, the excessive accumulation of preservatives in the human body is a relatively common issue, resulting from impaired metabolic elimination (Paula Neto et al., 2017). This underscores the importance of implementing biomonitoring practices, particularly given the potential of these substances to induce cytogenotoxic effects. Also, these substances, after going through detoxifying metabolic reactions through enzymatic activities, may generate toxic intermediates, such as free radicals and reactive oxygen species that may lead to primary DNA lesions (Costa et al., 2006). The fundamental issue associated with the formation of these lesions is that, if not adequately repaired, they can become incorporated into the genome, resulting in mutations in somatic and germ cells that subsequently increase the risk of cancer and may affect future generations (Bonassi et al., 2004; Heuser et al., 2007;). For this reason, as previously mentioned, conducting monitoring assays is crucial for the early diagnosis of cancer. To assess genotoxicity, various tests can be employed, such as the micronucleus test, comet assay, sister chromatid exchange assay, and chromosome aberration test, each with its specific endpoints and advantages (Pinto et al., 2024). Regarding cytotoxicity, many tests have been proposed in the literature such as cellular viability, MTT assay, PCE/NCE ratio and others. 

Recent studies have advanced our understanding of how food preservatives interact with cellular pathways, especially concerning oxidative stress, DNA repair inhibition, and epigenetic alterations (Zamora et al., 2022). Modern techniques, such as high-throughput genotoxicity screening and omics approaches, have been increasingly adopted to evaluate the safety of food additives with greater precision and relevance to human health (Mahato et al., 2024). These developments reinforce the need to continually reassess regulatory thresholds and public health policies regarding dietary exposure to food preservatives.

Considering the global increase in the consumption of processed foods, this systematic review aims to address the following question: can food preservatives indeed induce cytogenotoxicity? Additionally, this study seeks to evaluate the quality of research conducted in this field to determine the reliability of the conclusions obtained for future studies. 

## Material and Methods

### Eligibility criteria

This systematic review adhered to the 2020 PRISMA guidelines for reporting. We applied the PICOS framework as follows: P (Cells), I (Food preservative), C (Control group), O (Cytogenotoxicity).

Studies were included in our analyses if they met the following criteria: 1) studies measuring genetic damage and/or cellular death; 2) published in English; 3) studies that provided data in accordance with recognized scientific standards. Studies were excluded from analyses if they met the following criteria: 1) conference abstracts, reviews, editorials, and letters; 2) full-text not available in English; 3) studies with unavailable data/ un-extractable data; 4) studies using non-mammalian cells; 5) studies that did not measure genotoxicity and/or cytotoxicity 6) studies with incomplete or unclear results.

### Data search

A comprehensive search was conducted in March 2025, across PubMed, SCOPUS, and Web of Science to find relevant articles. The full search string used for all databases, based on a combination of keywords and Boolean operators, was as follows: “(Food preservative) OR (Preservative) AND (DNA Damages) OR (Damage, DNA) OR (Damages, DNA) OR (DNA Injury) OR (DNA Injuries) OR (Injuries, DNA) OR (Injury, DNA) OR (Genotoxic Stress) OR (Genotoxic Stresses) OR (Stresses, Genotoxic) OR (Genotoxicity) OR (Mutagenicity) OR (Comet assay) OR (Micronucleus assay) OR (Sister chromatid exchange) OR (Chromosomal aberration test) OR (Stresses, Genotoxic) (Micronucleus) OR (Micronucleated cell) OR (Chromosome damage) OR (Chromosomal injury) OR (Chromosome breakage) OR (Chromosome aberration test) OR (Cytotoxicity) OR (Cellular death), OR (Cell death) OR (Death) OR (Dying cells) OR (Cell dye) OR (Cellular viability) AND (Mammalian) OR (Mammalian cells).” We also conducted a thorough manual search for references and related articles. To ensure our search terms were effective, we verified that they yielded a diverse selection of pertinent studies. The search was not limited to publication dates. Three reviewers (TGP, DVS, and DAR) independently screened the abstracts. Full-text evaluations were carried out to establish eligibility, and any discrepancies among the reviewers were addressed through discussion until a consensus was achieved.

### Data extraction and quality assessment 

Three reviewers (TGP, DVS, and DAR) independently extracted data from eligible studies by examining titles, abstracts, and full texts, resolving any disagreements through discussion. The relevant data were organized, which includes the following details: authors, publication year and country, cell types, exposure duration, assay conducted, number of cells evaluated, geno- and cytotoxicity assays utilized, blind analysis status, statistical methods, negative control, and key findings.

The quality of the included studies was independently assessed by three reviewers (TGP, DVS, and DAR), with all relevant variables (confounders) considered in the evaluation. In terms of the methodology for classifying the studies, those in which up to one confounder was not controlled were rated as Strong, and studies with two uncontrolled confounders were categorized as Moderate and the studies with three or more uncontrolled confounders were consideres Weak at final rating, as described elsewhere (Pinto et al., 2024).

## Results

### Study selection

**Figure 1-:**
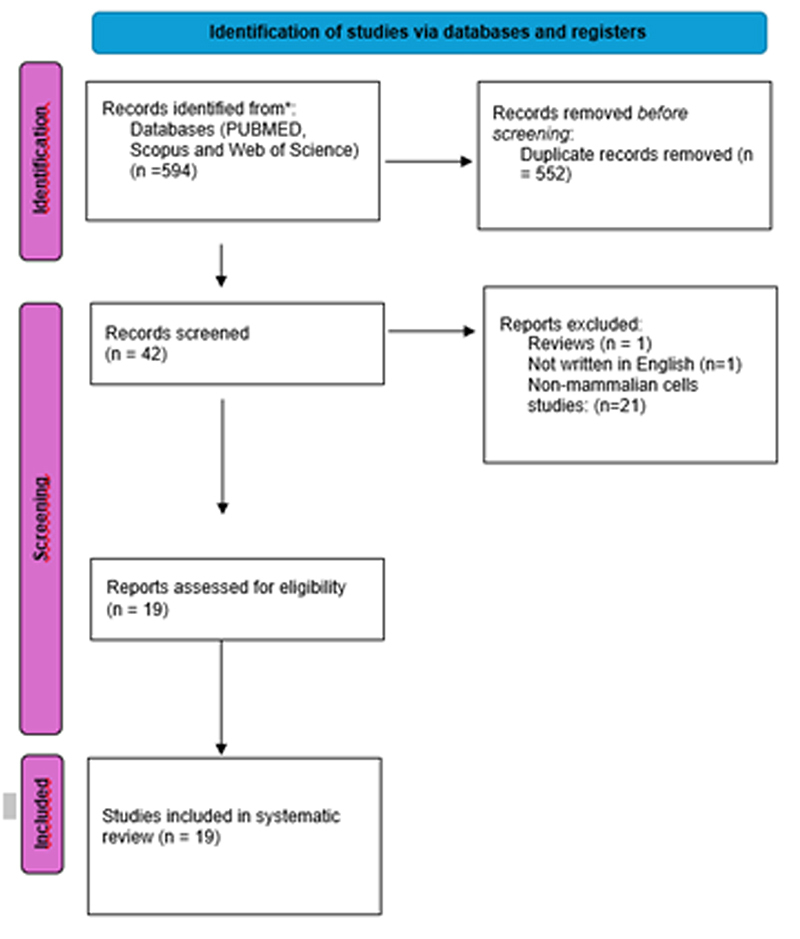
Flow chart of the study.

The initial online search yielded 594 scientific records; however, 552 of these were duplicates and, thus, excluded. Following an assessment of the titles and abstracts, 23 studies were deemed irrelevant for the purposes of this research and were removed. This exclusion applied to reviews, case reports, commentaries, editorials, non-English papers, and letters to the editor. Full manuscripts from 19 studies were carefully read by the authors of this article. The flow chart of the study is presented in Figure 1.

### General characteristics of the included studies

**Figure 2-:**
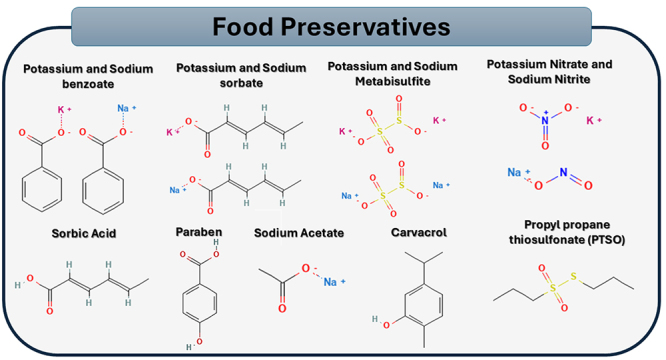
Chemical structure of food preservatives investigated in this study.

The most important characteristics of the evaluated studies can be visualized in Table 1. A total of 19 studies were evaluated, being seven conducted in Türkiye, two in Spain and one in each of the following countries: China, Iran, Egypt, Thailand, Brazil, France, Greece, United Kingdom, Germany, and Romania. Different food preservatives were investigated, such as sodium benzoate, sodium sorbate, butylated hydroxyanisole, butylated hydroxytoluene, sodium nitrite, sodium nitrate and sorbid acid (Figure 2). The year of publication found in the articles ranged between 1987 and 2018. Table 1 includes such data.

**Table 1-:** The most important characteristics of the studies included in this systematic review.

Author	Year of publication	Country	Investigated conservative
Altunkaynak and Avuloglu-Yilmaz	2024	Türkiye	Sodium acetate Sodium sulfite
Fang *et al.*	2024	China	Glycerol monocaprylate (GMC)
Ali *et al.*	2018	Egypt	Sodium benzoate
Mohammadzadeh-Aghdash *et al.*	2018	Iran	Sodium acetate Sodium diacetate Potassium sorbate
Güzel Bayülken *et al.*	2018	Türkiye	Paraben esters
Güzel Bayülken *et al.*	2017	Türkiye	Paraben
Llana-Ruiz-Cabello *et al.*	2016	Spain	Carvacrol
Mellado-García *et al.*	2016	Spain	Propyl thiosulphinate oxide (PTSO)
Pongsavee *et al.*	2015	Thailand	Sodium benzoate
Mamur *et al.*	2012	Türkiye	Sodium sorbate
Carvalho *et al.*	2011	Brazil	Sodium metabisulfite
Zengin *et al.*	2011	Türkiye	Sodium benzoate Potassium benzoate
Mamur *et al.*	2010	Türkiye	Potassium sorbate
Mpountoukas *et al.*	2008	Greece	Potassium Sorbate Potassium nitrate Sodium benzoate
Yavuz-Kocaman *et al.*	2008	Türkiye	Potassium Metabisulfite
Fontana *et al.*	2001	France	Sodium nitrite
Ferrand *et al.*	2000	United Kingdom	Sorbic acid amine reaction products
Jung *et al.*	1992	Germany	Sorbic Acid Potassium Sorbate
Luca *et al.*	1987	Romania	Sodium Nitrite

### Variables related to food preservative exposure and genotoxicity

The analysis of 19 articles revealed a variety of assays employed to assess genotoxicity. More specifically, 12 employed the micronucleus assay, analyzing a minimum of 1,000 cells (Table S1). The tissues assessed included bone marrow, liver, and human peripheral blood lymphocytes. Nine studies conducted the comet assay, evaluating between 50 and 100 cells per test, primarily focusing on bone marrow and liver tissues. Seven studies assessed chromosomal aberrations (CA), examining 100 to 600 metaphases across various tissues, including bone marrow and liver. Additionally, four studies evaluated sister chromatid exchanges (SCE), analyzing between 50 and 100 metaphases from tissues such as bone marrow and human lymphocytes (Table S1). Notably, 16 out of the 19 studies that assessed genotoxicity (including MN, comet, SCE, and CA) also performed cytotoxicity tests, such as cell viability assays, emphasizing the importance of correlating DNA damage with cellular death.

Regarding methodological rigor, five studies explicitly mentioned the carrying out of blind analyses, whereas 14 either did not report this information or did not perform such analyses. Control groups were present in all studies, ensuring appropriate comparisons to validate experimental results. Statistical analyses were applied in all studies, predominantly using ANOVA for significance testing. It is noteworthy that tissue evaluation varied significantly, as follows: bone marrow, liver, and human peripheral blood lymphocytes, with others examining tissues such as bladder and umbilical vein endothelial cells (Table S1).

## Results

Out of the 19 studies reviewed, 13 detected genotoxicity related to various substances, including sodium benzoate, potassium sorbate, and sodium metabisulfite, which consistently induced micronucleus formation and chromosomal abnormalities. Additionally, 15 out of the 19 studies reported cytotoxicity, indicated by reductions in the proliferation index and cell viability. Conversely, five studies did not observe any statistically significant differences in genotoxicity or cytotoxicity. This highlights a substantial concern regarding the genotoxic and cytotoxic effects of certain food preservatives, warranting further investigation and regulatory scrutiny (Table S2).

Genotoxicity findings were primarily based on assays, such as micronucleus formation, chromosomal aberrations, DNA strand breaks, and sister chromatid exchanges, which consistently showed increased DNA damage after exposure to these preservatives. For example, sodium benzoate repeatedly induced micronucleus formation and chromosomal abnormalities, as documented by Zengin et al. (2011) and Pongsavee (2015) in lymphocytes at varying concentrations. Similar genotoxic effects, including elevated chromosomal aberrations and DNA strand breaks, were reported for potassium sorbate and sodium metabisulfite across several studies.

Cytotoxicity was observed in most studies, assessed through reductions in cell viability, proliferation index, and mitotic index, often showing a dose-dependent pattern. Ali et al. (2020) reported DNA fragmentation associated with increasing concentrations of preservatives, while Güzel Bayülken et al. (2017) and Mamur et al. (2010) found significant decreases in cell viability following exposure to parabens and sodium sorbate, respectively.

Overall, the integrated results from multiple assay types consistently demonstrate the genotoxic and cytotoxic potential of various food preservatives, underscoring the need for careful safety evaluation in both food and pharmaceutical applications (Table S2).

### Quality assessment

The quality assessment of the articles indicated a robust foundation for the findings presented in Table 2. Out of the 19 articles reviewed, 15 were rated as Strong, demonstrating rigorous methodologies, including blind analysis and thorough statistical descriptions. Three articles were rated as Moderate, primarily due to the inclusion of fewer confounders or limited parameters assessed, while only one article received a Weak rating due to multiple confounders considered. This distribution of quality ratings suggests that the majority of studies are trustworthy, lending credibility to the overall conclusions drawn regarding mutagenicity and cytotoxicity.

**Table 2-:** Study quality and final ratings.

Author	Number ofconfounders	Missing details (confounders)	Rating
Altunkaynak and Avuloglu-Yilmaz, 2024	1	Blind analysis	Strong
Fang et al., 2024	1	Blind analysis	Strong
Ali et al., 2020	1	Blind analysis	Strong
Mohammadzadeh-Aghdash et al., 2018	1	Blind analysis	Strong
Güzel Bayülken et al., 2018	1	Blind analysis	Strong
Güzel Bayülken et al., 2017	2	Blind analysis Comet assay parameter	Moderate
Llana-Ruiz-Cabello et al., 2016	1	Blind analysis	Strong
Mellado-García et al., 2016	-	-	Strong
Pongsavee, 2015	4	Nº of cells evaluated Blind Analysis Statistical description Cytotoxicity	Weak
Mamur et al., 2012	1	Blind analysis	Strong
Carvalho et al., 2011	1	Comet assay parameter	Strong
Zengin et al., 2011	2	Blind analysis Comet assay parameter	Moderate
Mamur et al., 2010	1	Blind analysis	Strong
Mpountoukas et al., 2008	-	-	Strong
Yavuz-Kocaman et al., 2008	1	Blind analysis	Strong
Fontana et al., 2001	1	Cytotoxicity	Strong
Ferrand et al., 2000	2	Blind analysis Cytotoxicity	Moderate
Jung et al., 1992	1	Cytotoxicity	Strong
Luca *et al.,* 1987	-	-	Strong

## Discussion

The objective of this study was to evaluate the cytogenotoxicity of various food preservatives through a systematic analysis of 19 articles. To the best of our knowledge, the approach has not been addressed so far. 

At this present, it is essential to address the nomenclature of preservatives and food additives in this article to prevent confusion and ensure a precise understanding of their distinct roles and functions. The present article focuses on preservatives, which are substances added to food to prevent spoilage and extend shelf life by inhibiting microbial growth (Awuchi et al., 2020). Although all preservatives qualify as food additives, not all food additives function as preservatives. Food additives may include flavor enhancers, colorings, and stabilizers, which enhance the sensory qualities of food but do not necessarily prevent spoilage (Awuchi *et al.*, 2020). Food preservatives, on the other hand, specifically focus on preservation, assuring safety and quality by efficiently prolonging the product’s usability without losing its integrity (Awuchi *et al.*, 2020).

The findings revealed significant concerns regarding the genotoxic effects of substances, such as sodium benzoate, potassium sorbate, and sodium metabisulfite, with many studies demonstrating their capacity to induce micronucleus formation, DNA breakage and chromosomal abnormalities. Notably, sodium benzoate was frequently associated with these adverse effects across multiple studies, prompting calls for increased regulatory scrutiny. Additionally, cytotoxicity was reported in most of the studies, with evidence of reduced cell viability and proliferation in response to these compounds. A comparative analysis of these results across the studies showed consistent cytogenotoxic findings in *in vitro* models; however, three studies (Fontana et al., 2001; Yavuz-Kocaman et al., 2008; Carvalho et al., 2011) showed no statistically significant effects at lower concentrations, highlighting variability related to dosage, exposure duration, and assay sensitivity. This comprehensive review highlights the need for further investigation into the safety of commonly used food preservatives.

It is important to stress that the genotoxicity induced by food preservatives, such as sodium benzoate and potassium sorbate, is frequently associated with the formation of reactive oxygen species (ROS) (Walczak-Nowicka and Herbet, 2022). These reactive species can directly damage DNA, causing single- and double-strand breaks and forming adducts with nitrogenous bases (Chatterjee and Walke, 2017). Additionally, the genotoxicity is not limited to DNA. The interaction of reactive species with proteins and lipids can also trigger a cascade of events that contribute to cellular toxicity (Liguori et al., 2018). Nevertheless, the magnitude of this damage is modulated by the efficiency of cellular DNA repair mechanisms, which may become overwhelmed under prolonged exposure or at high concentrations of preservatives (Maynard et al., 2009). While these mechanisms are well described, their relative contribution across different preservatives and experimental systems remains to be clarified. Future studies should directly compare ROS levels, repair pathway activation, and apoptosis signaling among preservatives using standardized protocols.

On the other hand, the analysis of the genotoxic effects associated with food preservatives highlights the importance of a careful evaluation of the dose-response relationship. While several studies have reported significant induction of micronucleus formation and chromosomal aberrations at high concentrations, the extrapolation of these findings to typical human exposure levels remains limited (Luca *et al.*, 1987; Fontana et al., 2001; Yavuz-Kocaman et al., 2008; Carvalho et al., 2011; Zengin et al., 2011; Güzel *et al.*, 2017). This gap is considered critical to determine whether the doses used in experimental studies accurately reflect human exposure levels and whether they fall below the limits considered safe for daily consumption. In terms of regulatory relevance, it is important to note that many of the reviewed studies employed concentrations significantly higher than the Acceptable Daily Intake (ADI) limits established by International Regulatory Agencies. For instance, the ADI for sodium benzoate set by JECFA is 0-5 mg/kg bw/day (World Health Organization - WHO, 2000). Yet, several studies tested concentrations above 500 µg/mL, levels unlikely to be reached through normal dietary exposure. This discrepancy highlights the need for further research using physiologically relevant concentrations to improve translational validity. Anyway, the importance of these findings extends beyond academic interest, as they have direct implications for public health and food safety regulations (Vandenberg and Bugos, 2021). The documented genotoxic and cytotoxic effects of widely consumed food preservatives raise concerns about their long-term impact on human health, particularly when consumed in high doses or over extended periods (Warner, 2024). Regulatory bodies may need to re-evaluate the permissible levels of these preservatives in food products to safeguard consumer health. Moreover, the protective effects observed with certain combinations, such as sodium benzoate with royal jelly, suggest potential avenues for developing safer food preservation methods (Acar, 2021). In fact, other antioxidants, such as vitamin C, vitamin E, curcumin, and flavonoids have shown protective effects against chemically induced genotoxicity and should be further investigated as dietary co-factors or food formulation agents (Singh et al., 2016). These findings underscore the relevance of ongoing research in informing regulatory policies and guiding industry practices to mitigate risks associated with food preservatives. The identification of compounds that can mitigate the genotoxic effects of food preservatives represents a promising area for future research, which needed to evaluate the efficacy of other natural antioxidants. Additionally, adjustments in food manufacturing processes, such as reducing preservative concentrations or substituting them with natural alternatives, should be considered to ensure consumer safety without compromising product quality

Furthermore, assessing the quality and trustworthiness of the key findings is crucial for drawing reliable conclusions. The review identified a range of methodologies, with common techniques as for example the micronucleus and comet assays employed to evaluate genotoxicity. Nevertheless, while all studies included control groups, only five explicitly described blind analyses, indicating variability in experimental rigor (Luca *et al.*, 1987; Jung et al., 1992; Carvalho et al., 2011; Mellado-García et al., 2016). The use of one-way ANOVA for data analysis across most studies enhances the robustness of the findings, although variations in sample sizes and study designs may introduce biases. 

Taking into consideration that micronuclei are infrequent in normal dividing cells and that their occurrence significantly increases after exposure to systemic clastogens and aneugens, it is justifiable to evaluate a minimum number of cells to ensure reliable genotoxicity results. In this context, it is reasonable to assert that the total number of cells assessed in this analysis did not affect the quality of the mutagenicity data, regardless of the target tissue (Bonassi et al., 2004; Igl et al., 2019). The minimum sample sizes were similarly justified for other genotoxicity assays, including the requirement of at least 50 evaluated cells for the comet assay, as outlined by the comet assay expert group (Møller et al., 2020).

In terms of limitations, some inherent issues should be considered. The variability in experimental designs, including differences in cell models, exposure durations, and outcome measurements, introduces heterogeneity that can affect direct comparisons between studies. Moreover, the exclusive reliance on *in vitro* assays, while valuable for mechanistic insights, limits the direct extrapolation of results to human health outcomes due to the complexity of *in vivo* metabolism and systemic interactions. Future research integrating *in vivo* studies and standardized methodologies will further strengthen the evidence base and support more definitive risk assessments.

**Figure 3 -:**
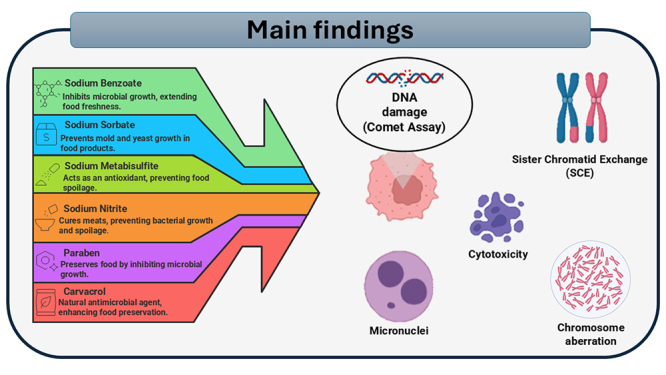
Most food preservatives demonstrate cytotoxicity in mammalian cells.

In conclusion, the systematic analysis of 19 articles indicates a concerning link between certain food preservatives and genotoxicity, as demonstrated by the fact that most studies explicitly reported cytogenotoxic effects related to these agents in mammalian cells (Figure 3). This substantial proportion underscores the potential risk posed by substances such as sodium benzoate, potassium sorbate, and sodium metabisulfite. Among these studies, 18 out of 19 were classified as Strong or Moderate based on our methodology, which includes multiple analyses of confounders. This significantly enhances the credibility of the findings and underscores the necessity for increased scrutiny of these preservatives in food products. As the demand for transparency in food labeling increases, the findings advocate more rigorous testing and monitoring of food preservatives. Consumers have the right to be informed about the substances they ingest, and the scientific community must continue to investigate the safety of these compounds diligently. This study serves as a call to action for researchers, regulators, and consumers alike to prioritize health and safety in the context of food consumption.

## Supplementary material

The following supplementary material is available for this article:

Table S1 - Variables of the reviewed studies related to food preservative exposure and cytogenotoxicity.

Table S2 - Summary of key findings from studies organized by publishment chronology.

## Data Availability

 There is no data sharing available for this article.
