# Supplementary material for: Cytogenotoxicity of food preservatives in mammalian cells: A systematic review
Source: Genet Mol Biol. 2025 Dec 15;49(Suppl 1):e20250137. doi: 10.1590/1678-4685-GMB-2025-0137 (PMC12766372; doi:10.1590/1678-4685-GMB-2025-0137)
Supplement: Table S1 - [file 1415-4757-GMB-49-s1-e20250137-s1.pdf]

Genetics and Molecular Biology
https://doi.org/10.1590/1678-4685-GMB-2025-0137

Supplementary Material to "Cytogenotoxicity of food preservatives in mammalian cells: A systematic review"

Table S1 - Variables of the reviewed studies related to food preservative exposure and cytogenotoxicity.

Author	Cell type	N	Gender	Age	Genotoxicity assay	N. of evaluated units	Stain	Evaluated parameters	Inclusion criteria	Cyto-toxicity analysis	Blind analysis	Proper statistical description	Control group
Avuloğlu-Yılmaz et al. 2024	The human hepatocellular carcinoma (HepG2) cell line And Human peripheral blood	For Comet assay: five concentrations of sodium acetate-NaA (15.63, 31.25, 62.50, 125, and 250 µg/mL) and sodium sulfite - NaS (3.91, 7.81, 15.63, 31.25, and 62.50 µg/mL)	1 male, 1 female	22 – 27 years	Comet Assay	200	Ethidium bromide	tail intensity (%), tail length (µm) and tail moment)	Yes	Yes (MTT And Trypan Blue/Comet Assay)	No	Yes (analyzed by one-way ANOVA followed by Dunnet's multiple comparison tests)	Yes
Fang et al. 2024	Strains of Salmonella typhimurium (TA97, TA98, TA100, and TA1535) and Chinese hamster ovary (CHO) cell	For Bacterial reverse mutation (Ames) test: glycerol monocaprylate (GMC), solubilized in dimethyl sulfoxide (DMSO), at concentrations of 21, 62, 185, 556, 1667, 5000 µg/plate.<br><br>For Chromosome aberration (CA) test: glycerol monocaprylate (GMC) at concentrations of 0.16, 0.31, and 0.62 mg/ mL	-	-	Bacterial reverse mutation (Ames) test<br><br>Chromosome aberration (CA) test	300 metaphase (Chromosomal aberration)	Giemsa (CA)	Cell and colony counting	-	Yes (Relative increase in cell count after S9 activation)	No	Yes (analyzed by one-way analysis of variance - ANOVA)	Yes
Ali et al. 2018	Hepatocytes (liver) and bone marrow from albino rats	35 (n= 5 per group)<br><br>Group 1 (Control): water<br><br>Group 2: 5 mg/kg of SY and 10 mg/kg of NaB.<br><br>Group 3: 5 mg/kg of SY and 100 mg/kg of NaB.<br><br>Group 4: 50 mg/kg of SY and 100 mg/kg of NaB.<br><br>Group 5: 50 mg/kg of SY and 10 mg/kg of NaB.<br><br>Group 6: 200 mg/kg of SY and 750 mg/kg of NaB.<br><br>Grupo 7: 20 mg/kg of SY and 75 mg/kg of NaB.	Female	8 - 10 weeks old	Chromosomal aberration (CA) assay<br><br>Alkaline Comet Assay	600 metaphase (chromosomal aberration)<br><br>50–100 randomly selected cells (Alkaline Comet Assay)	Chromosomal aberration (Giemsa)<br><br>Alkaline Comet Assay (ethidium bromide)	Cell Counting (chromosomal aberration)<br><br>Tail length, tail DNA%, tailed % and tail moment units (Alkaline Comet Assay)	-	Yes<br><br>(Fragmentation of liver DNA)	No	Yes (analyzed by one way ANOVA followed by student's t-test)	Yes
Mohammadzadeh-Aghdash et al. 2018	human umbilical vein endothelial cells (HUVECs)	concentration range (25, 50, 100, and 200µM) (SA, SDA, PS)<br><br>Negative Control: untreated cells	-	-	DNA ladder assay	-	-	percentage of DNA	-	Yes<br><br>(MTT assay and Dapi staining assay)	No	Yes (analyzed by one-way and two-way analyses of variance (ANOVA))	Yes

Author	Cell type	N	Gender	Age	Genotoxicity assay	N. of evaluated units	Stain	Evaluated parameters	Inclusion criteria	Cyto-toxicity analysis	Blind analysis	Proper statistical description	Control group
		Positive control: DMSO-treated cells.											
Guzel Bayülken et al. 2018	Human peripheral lymphocytes	2000 (1000 cells per donor)	1 male, 1 female	25-30 years	Cytokinesis-block micronucleus (CBMN) assay	2000 (1000 cells per concentration)	Giemsa	Celll count	Yes	Yes (CBPI test used)	No	ANOVA followed by Dunnett test for multiple comparisons, mean ± SD calculated	Yes
Güzel Bayülken et al. 2017	Cell culture from human peripheral blood	Negative Control: Untreated cells.<br><br>Solvent Control: Cells treated with DMSO (dimethylsulfoxide).<br><br>Positive Control: cells treated with Mitomycin C (MMC) or hydrogen peroxide (H_2O_2).<br><br>Paraben Treatment Groups: cells treated with different concentrations of paraben (50, 100, 250, 500 and 1000 µg/mL) for different periods (24 and 48 hours).	-	-	Cytokinesis-block micronucleus (CBMN) Assay<br><br>Chromosome aberration (CA)<br><br>Sister chromatid exchange (SCE) Assay<br><br>Alkaline Comet Assay	1000 cells (CBMN)<br><br>100 metaphases (CA)<br><br>50 metaphase (SCE)<br><br>100 randomly selected cells (Alkaline Comet Assay)	Cytokinesis-block micronucleus (CBMN) Assay, Chromosome aberration (CA) and Sister chromatid exchange (SCE) Assay (Giemsa)<br><br>Alkaline Comet Assay (ethidium bromide)	Cell Counting (CBMN, CA and SCE)<br><br>Tail length (Alkaline Comet Assay)	Yes (donors = aged 25–30 years, non smoker,with no medical record of any chronic or acute disease)	Yes<br><br>Proliferation index (PI)<br><br>Mitotic Index (MI)<br><br>Cytokinesis-block proliferation index (CBPI)	No	Yes (analyzed by one-way analysis of variance (ANOVA) followed by Dunnett test)	Yes
Llana-Ruiz-Cabello et al. 2016	Bone marrow, stomach and liver from Wistar rats: strain RjHan:WI (type outbred rats)	Negative Control Group (n=10): treated with corn oil.<br><br>Positive Control Group (n=6): rats exposed to 200 mg/kg bw of ethyl methanesulfonate (EMS).<br><br>Experimental Group 1 (n=10): exposed to 81 mg/kg bw of carvacrol.<br><br>Experimental Group 2 (n=10): exposed to 256 mg/kg bw of carvacrol.<br><br>Experimental Group 3 (n=10): exposed to 810 mg/kg bw of carvacrol.	23 male and 23 female rats	8 - 10 week-old	Micronucleus assay<br><br>Standard and enzyme-modified comet assay (Endo III and FPG sites)	500 (Micronucleus)<br><br>Not informed (Comet assay)	Micronucleus Assay (Giemsa)<br><br>Comet Assay (Not informed)	Cell counting (Micronucleus Assay)<br><br>percentage of DNA in tail (Comet Assay)	-	Yes PCE/NCE ratio	No	Yes<br><br>Micronucleus (analyzed by analysis of variance (ANOVA) followed by Dunnett's multiple comparison tests)<br><br>Comet Assay (analyzed by non-parametric KruskaleWallis test followed by the Mann-Whitney U test)	Yes
Mellado-García et al. 2016	Bone marrow, stomach and liver from Wistar rats, strain RjHan:WI (type outbred rats)	Negative Control Group (n=10): water with tween 20 (vehicle)<br><br>Positive Control Group (n=6): rats exposed to 200 mg/kg bw of ethylmethanesulfonate (EMS) Experimental Group 1 (n=10): exposed to 55 mg/kg bw of PTSO.<br><br>Experimental Group 2 (n=10): exposed to 17,4 mg/kg bw of PTSO.<br><br>Experimental Group 3 (n=10):	23 male and 23 female rats	8 week-old	Micronucleus assay<br><br>Standard and enzyme-modified comet assay (Endo III and FPG sites)	500 (Micronucleus)<br><br>100 (Comet assay)	Micronucleus Assay (Giemsa)<br><br>Comet Assay (SYBR Gold)	Cell counting (Micronucleus Assay)<br><br>percentage of DNA in tail (Comet Assay)	-	Yes PCE/NCE ratio	Yes (MN)	Yes The statistical approach included the one-way ANOVA followed by the Tukey-Kramer's test, which was used to evaluate the significance of the differences in% MN and comet assay. Significant differences of PCE/total were calculated by the Kruskal-Wallis Test (non-parametric ANOVA).	Yes

Author	Cell type	N	Gender	Age	Genotoxicity assay	N. of evaluated units	Stain	Evaluated parameters	Inclusion criteria	Cyto-toxicity analysis	Blind analysis	Proper statistical description	Control group
		exposed to 5,5 mg/kg bw of PTSO.											
Pongsavee et al. 2015	human lymphocyte cell line	Sodium benzoate concentrations: 0.5, 1.0, 1.5 and 2.0 mg/mL<br><br>Control Group: untreated cells	-	-	Micronucleus Assay<br><br>Chromosome breakage	Not informed	Micronucleus Assay<br><br>and<br><br>Chromosome breakage<br><br>(Giemsa)	Cell Counting	-	No	No	No<br>(ANOVA)	Yes
Mamur et al. 2012	Human peripheral blood lymphocytes	Negative Control: Sterile distilled water.<br><br>Positive Control: Mitomycin-C (MMC) for cultured lymphocytes and H2O2 for isolated lymphocytes.<br><br>Sodium sorbate (SS) Treatment: four different concentrations (100, 200, 400 and 800 µg/ml).	-	-	Micronucleus assay<br>(MN)<br><br>Comet Assay<br>(SCGE)<br><br>Chromosomal aberrations<br>(CA)<br><br>Sister chromatid exchange assay<br>(SCE)	2000<br>(MN)<br><br>200<br>(SCGE)<br><br>200 metaphase<br>(CA)<br><br>50 metaphase<br>(SCE)	Giemsa<br>(MN)<br><br>Not informed<br>(SCGE)<br><br>Giemsa<br>(CA)<br><br>FPG (fluorescence plus Giemsa)<br>(SCE)	Cell counting<br>(MN / CA / SCE)<br><br>Tail intensity<br>(SCGE)	Yes	Cytokinesis-block proliferation index (CBPI)<br>(MN)<br><br>Trypan Blue<br>(SCGE)	No	Yes<br>(Z test, correlation and regression)	Yes
Carvalho et al. 2011	Blood, liver and bone marrow from Outbred CF1 mice	Negative control: water.<br><br>Positive control: cyclophosphamide (25 mg/kg). Groups treated with SMB: doses of 0.5 g/kg, 1 g/kg and 2 g/kg.	25 male and 25 female rats	5-7 weeks-old	Micronucleus assay (MN)<br><br>Alkaline Comet Assay	1000<br>(Micronucleus)	Giemsa<br>(Micronucleus)<br><br>Ammonium nitrate, silver nitrate, tungstosilicic acid, formaldehyde and sodium carbonate<br>(Comet Assay)	Cell counting<br>(MN)<br><br>Length tails = score<br>(Comet Assay)	-	Yes<br>PCE/NCE ratio	Yes	Yes<br>(Non-parametric two-tailed Kruskal–Wallis Test with the Dunn correction)	Yes
Zengin et al. 2011	Human peripheral blood lymphocytes	Negative and Positive Controls: negative (no treatment) and positive (mitomycin-C)<br><br>Sodium benzoate (SB) treatment: five different concentrations (6.25, 12.5, 25, 50 and 100 µg/ml) for 24 and 48 hours.<br><br>Potassium benzoate (PB) treatment: five different concentrations of PB (62.5, 125, 250, 500 and 1000 µg/ml) for 24 and 48 hours.	-	-	Micronucleus assay<br>(MN)<br><br>Comet Assay<br>(SCGE)<br><br>Chromosomal aberrations<br>(CA)<br><br>Sister chromatid exchange assay<br>(SCE)	2000<br>(MN)<br><br>200<br>(SCGE)<br><br>200 metaphase<br>(CA)<br><br>50 metaphase<br>(SCE)	Not informed<br>(MN)<br><br>Not informed<br>(SCGE)<br><br>Not informed<br>(CA)<br><br>Giemsa<br>(SCE)	Cell counting<br>(MN / CA / SCE)<br><br>Tail moment<br>(SCGE)	Yes	Cytokinesis-block proliferation index (CBPI)<br>(MN)<br><br>Trypan Blue<br>(SCGE)	No	Yes<br>(Z test, correlation and regression)	Yes
Mamur et al. 2010	Human peripheral blood lymphocytes	Negative Control: Sterile distilled water.<br><br>Positive Control: Mitomycin-C (MMC) for cultured lymphocytes and H2O2 for isolated lymphocytes.	-	-	Micronucleus assay<br>(MN)<br><br>Comet Assay<br>(SCGE)<br><br>Chromosomal aberrations	1000<br>(MN)<br><br>200<br>(SCGE)<br><br>100 metaphase<br>(CA)	Giemsa<br>(MN)<br><br>Ethidium bromide<br>(SCGE)<br><br>Giemsa<br>(CA)	Cell counting<br>(MN / CA / SCE)<br><br>Tail intensity<br>(SCGE)	Yes	Cytokinesis-block proliferation index (CBPI)<br>(MN)<br><br>Trypan Blue<br>(SCGE)	No	Yes<br>(Z test)	Yes

Author	Cell type	N	Gender	Age	Genotoxicity assay	N. of evaluated units	Stain	Evaluated parameters	Inclusion criteria	Cyto-toxicity analysis	Blind analysis	Proper statistical description	Control group
		Potassium sorbate Treatment: four different concentrations (125, 250, 500 and 1000 µg/ml).			(CA)<br><br>Sister chromatid exchange assay (SCE)	50 metaphase (SCE)	Giemsa (SCE)						
Mpountoukas et al. 2008	Human whole blood	Potassium sorbate, potassium nitrate and, sodium benzoate Treatment: six different concentrations (0 (control), 8, 4, 2, 0.2 and, 0.02 mM).	2 males and 4 females	20 - 30 years old	Sister chromatid exchange assay (SCE)	200 metaphases (SCE)	Fluorescence plus Giemsa (FPG) technique	Cell counting	Yes	Yes<br>Mitotic Index (MI)<br>Proliferation rate index (PRI)	Yes	Yes<br>(ANOVA and Chi-square-test)	Yes
Yavuz-Kocaman et al. 2008	Human whole blood<br><br>and<br><br>Bone marrow (Rattus norvegicus var. albino)	Potassium metabisulfite Treatment: four different concentrations (25, 50, 100, and 200 µg/ml).<br><br>Negative control (BrdU, 10 µg/ml)<br><br>Positive control: ethyl methanesulfonate (EMS - 125 µg/ml)<br><br>Rat exposure: intraperitoneally treated with three different concentrations (150, 300, and 600 mg/kg b.w.)	2 male and 2 female (Human)<br><br>2 male and 2 female (Rat)	22 - 23 years old (Human)<br><br>12 - 16 weeks-old (Rat)	Micronucleus assay (MN)<br><br>Chromosomal aberrations (CA)<br><br>Sister chromatid exchange assay (SCE)	1000 (MN)<br><br>100 metaphase (CA)<br><br>100 metaphase (SCE)	Giemsa (MN)<br><br>Giemsa (CA)<br><br>Modified fluorescence plus Giemsa method (SCE)	Cell counting (MN / CA / SCE)	Yes	Yes<br>Mitotic Index (MI)	No	Yes<br>(T-test, orrelation and regression coefficients)	Yes
Fontana et al. 2001	Exfoliated cells from bladder	Control group: healthy females<br><br>Experimental group: occupational exposure (mineral jelly = sodium nitrite and N-phenyl-1-naphyhylamine)	76 Female	29 - 62 years old	Micronucleus Assay	1000	Propidium iodide	Cell counting	Yes	No	Yes	Yes<br>(Chi-square and MANCOVA)	Yes
Ferrand et al. 2000	HeLa cells	The cells were cultured in 96-well culture plates, with 10,000 cells seeded per well	-	-	3D test for DNA damage	-	-	Repair ratio (genomic and plasmid DNA)	Yes	No	No	ANOVA with Dunnett test	Yes (positive control)
Jung et al. 1992	Bone marrow from NMRI mice	Mice<br><br>Exposure at 500, 1500 and 5000 mg/kg body weight by oral administration of sorbic acid<br><br>negative control group, and a positive control group (gavage with cyclophos- phamide - 10 ml/kg body weight)<br>Potassium sorbate exposure: (400, 800 or 1200 mg/kg body weight)<br>N-nitrosodimethylamine (positive control; 5, 10 or 100mg/kg body weight) or the vehicle (negative control; 0.9 g NaCl/100 ml).	5 male and 5 female	Not informed	AMES<br><br>Micronucleus Assay (MN)<br><br>Sister chromatid exchange assay (SCE)<br><br>Unscheduled DNA Synthesis (UDS) and alkaline elution tests.	1000 (MN)<br><br>20 metaphase (SCE)	Giemsa (MN / SCE)	Cell counting	-	Yes	No	Yes<br>(Student's t-test and the one-way analysis of variance)	Yes
Luca et al. 1987	In vivo<br><br>Bone marrow from Wistar rats; Swiss mice; Chinchilla rabbits	In vivo<br>Control group: distilled water<br><br>Experimental: exposure to sodium nitrite at 1.72, 5.18, 15.55 and 46.66 mg/kg)	6 male rats<br>6 male mice<br><br>6 male rabbits	Wistar rats (8-10 weeks-old)<br><br>Swiss mice (10-12 weeks-old)	Chro- mosome aberration (CA)<br><br>Micronucleus Assay (MN)	50 metaphase (CA)<br><br>1000 (MN)	Stain not informed	Cell counting	-	Yes<br>PCE/NCE ratio	Yes	Yes<br>(chi- square)	Yes

Author	Cell type	N	Gender	Age	Genotoxicity assay	N. of evaluated units	Stain	Evaluated parameters	Inclusion criteria	Cyto-toxicity analysis	Blind analysis	Proper statistical description	Control group
	In vitro<br><br>BS-C1 and HeLa cells	In vitro<br><br>Control group: physiological saline<br><br>Experimental: exposure to sodium nitrite at 0.265 and 0.530 mg/ml		Chinchilla rabbits (30-32 weeks-old)									

MN = Micronucleus assay; **CA** = Chromosome aberrations; **SCE** = Sister Chromatid Exchange; **-- =** Not described; **N/A**= not applicable; **PCR-RFLP**= Real-time reverse transcription polymerase chain reaction – restriction fragment reverse; **C** = control group; **SY** = Sunset Yellow - food colorant (FCF, C16H10N2Na2O7S2 (dyecontent 90%); **NaB** = Sodium benzoate; **SA** = Sodium acetate; **SDA** = Sodium diacetate; **PS** = Potassium sorbate**DMSO** = Dimethylsulfoxide; **PTSO** = Propyl thiosulphinate oxide; **BHT** = Butylhydroxytoluene; **BHA** = Butylhydroxyanisole; **SA*** = Sorbic Acid; **PG** = Propyl Gallate; **SN** = Sodium Nitrate; **SMB** = Sodium metabisulfite.
