# Supplementary material for: Cytogenotoxicity of food preservatives in mammalian cells: A systematic review
Source: Genet Mol Biol. 2025 Dec 15;49(Suppl 1):e20250137. doi: 10.1590/1678-4685-GMB-2025-0137 (PMC12766372; doi:10.1590/1678-4685-GMB-2025-0137)
Supplement: Table S2 - [file 1415-4757-GMB-49-s1-e20250137-s2.pdf]

Genetics and Molecular Biology
https://doi.org/10.1590/1678-4685-GMB-2025-0137

Supplementary Material to "Cytogenotoxicity of food preservatives in mammalian cells: A systematic review"

Table S2 - Summary of key findings from studies organized by publishment chronology

Authors	Genotoxicity assay	Cytotoxicity assay	Genotoxicity Outcome	Cytotoxicity Outcome	Observation
Avuloğlu-Yılmaz et al. 2024	Increase in DNA damage (tail intensity, length, and moment) in human lymphocytes exposed to sodium acetate (from 31.25 µg/mL) and sodium sulfite (from 3.91 µg/mL)	Decrease in cell viability (MTT assay) in HepG2 cells only at the highest concentrations tested (2000 µg/mL for sodium acetate; 500 µg/mL for sodium sulfite)	Positive	Positive	-
Fang et al. 2024	No significant changes	No significant changes	Negative	Negative	-
Ali et al. 2018	Increase DNA damage (tail length) in liver DNA of animals in group 2 (5 mg/kg of SY + 10 mg/kg of NaB) and 4 (50 mg/kg of SY + 100 mg/kg of NaB).	The results demonstrated that the administration of these combinations (SY + NaB) resulted in a distinctive pattern of DNA fragmentation, indicative of	Positive	Positive	-

Authors	Genotoxicity assay	Cytotoxicity assay	Genotoxicity Outcome	Cytotoxicity Outcome	Observation
	Increase CA from group 6 (200mg SY + 750mg NaB), group 4 (50mg SY + 100mg NaB) and group 3 (5mg SY + 100mg NaB).	apoptosis. The degree of DNA fragmentation was found to be dose-dependent, with higher levels of SY and NaB resulting in greater fragmentation.			
Mohammadzadeh-Aghdash et al. 2018	No significant changes	No significant changes	Negative	Negative	-
Güzel Bayülken et al. 2018	Increased micronuclei at higher concentrations	-	Positive	-	Dose dependent relationship
Güzel Bayülken et al. 2017	Significant chromosomal aberrations were observed at concentrations of 500 and 250 µg/mL after 24 hours of exposure, suggesting clastogenic potential.<br><br>Only the highest concentration of parabens (1000 µg/mL) caused significant DNA migration, indicating DNA damage evaluated by Comet Assay.	Parabens caused a significant decrease in the proliferation index and mitotic index at all concentrations tested, indicating cytotoxic effects.	Positive	Positive	-
Llana-Ruiz-Cabello et al. 2016	No significant changes	Carvacrol increaase cytotoxicity in bone marrow	Negative	Positive	-

Authors	Genotoxicity assay	Cytotoxicity assay	Genotoxicity Outcome	Cytotoxicity Outcome	Observation
		(decrease PCE/NCE ratio in male rats from 256 mg/ bw and decrease PCE/NCE ratio in male and female rats from 810 mg bw)			
Mellado-García et al. 2016	No significant changes	No significant changes	Negative	Negative	-
Pongsavee 2015	Concentrations of 1.0, 1.5 and 2.0 mg/mL of sodium benzoate increased micronucleus formation in lymphocytes after 24 and 48 hours of incubation.<br><br>A concentration of 2.0 mg/mL sodium benzoate caused separation of sister chromatids and gaps in chromosomes after 24 and 48 hours.	Not Assessed	Positive	Not Assessed	-
Mamur et al. 2012	Sodium sorbate increased the frequency of CA at all concentrations tested (100, 200, 400 and 800 µg/ml) after 24 and 48 hours of treatment.	No significant changes for CBPI in Micronucleus Assay<br><br>Cell viability was greater than 97% by the trypan blue test.	Positive	Negative	For CA: aberrations included chromatid breaks, chromosome breaks, fragments, sister chromatid unions, chromatid exchanges and dicentric chromosomes.

Authors	Genotoxicity assay	Cytotoxicity assay	Genotoxicity Outcome	Cytotoxicity Outcome	Observation
	Sodium sorbate increased SCEs at concentrations of 400 and 800 µg/ml after 24 and 48 h of treatment.<br><br>Sodium sorbate increased the frequency of MN at concentrations of 400 and 800 µg/ml after 48 h of treatment.<br><br>Sodium sorbate caused DNA damage at all concentrations tested (100, 200, 400 and 800 µg/ml) after 1 hour of in vitro exposure.				
Carvalho et al. 2011	Exposure to 1 and 2g/kg of sodium metabisulfite (SMB) increased genotoxic damage in blood, liver and bone marrow cells.<br><br>Exposure to 2g/kg of sodium metabisulfite (SMB) increased micronuclei formation in blood and bone marrow cells.	SMB increase cytotoxicity in bone marrow (decrease PCE/NCE ratio after 2g/Kg exposure)	Positive	Positive	-

Authors	Genotoxicity assay	Cytotoxicity assay	Genotoxicity Outcome	Cytotoxicity Outcome	Observation
Zengin et al. 2011	Both SB and PB increased the frequency of CAand SCE at all concentrations and treatment periods.<br><br>Sodium benzoate (SB) increased the frequency of MN at concentrations of 25, 50 and 100 µg/mL.<br><br>Potassium benzoate (PB) increased the frequency of MN at concentrations of 125, 250, 500 and 1000 µg/mL.<br><br>Sodium benzoate increased genotoxicity at all concentrations tested (6.25, 12.5, 25, 50, and 100 µg/mL) by comet assay.	No significant changes for CBPI in Micronucleus Assay<br><br>Cell viability was greater than 98% by the trypan blue test.	Positive	Negative	For CA: chromatid and chromosome breaks were the most frequent aberrations observed.
Marmur et al. 2010	Potassium sorbate increased the frequency of CA at concentrations of 250, 500 and 1000 µg/ml, both after 24h and 48h of treatment.	No significant changes for CBPI in Micronucleus Assay<br><br>Cell viability was greater than 97% by the trypan blue test.	Positive	Negative	For CA: chromatid breaks were the most common aberrations.

Authors	Genotoxicity assay	Cytotoxicity assay	Genotoxicity Outcome	Cytotoxicity Outcome	Observation
	Potassium sorbate increased the frequency of SCE at all concentrations tested except 125 µg/ml after 24h of treatment.<br><br>Potassium sorbate increased genotoxicity at concentrations of 125, 250, 500 and 1000 µg/ml by comet assay.				
Mpountoukas et al. 2008	Potassium sorbate increased genotoxicity at concentrations of 8 mM by SCE<br><br>Sodium benzoate increased genotoxicity at concentrations of 4 and 8 mM by SCE	No significant changes	Positive	Negative	-
Yavuz-Kocaman et al. 2008	Potassium metabisulfite increased structural CA at all concentrations and time of exposure in cell culture of human blood.<br><br>Potassium metabisulfite increased the frequency of MN at all concentrations and time of exposure in cell culture of human blood.	Increase cytotoxicity at all concentrations and time of exposure in cell culture of human blood. (Mitotic Index (MI)<br><br>increase cytotoxicity at all concentrations after 12h of treatment and at the higher concentration (600	Positive	Positive	-

Authors	Genotoxicity assay	Cytotoxicity assay	Genotoxicity Outcome	Cytotoxicity Outcome	Observation
	Potassium metabisulfite increased structural CA at 300 and 600 µg/ml after 12h of treatment and after 24h at all concentrations tested in rat bone marrow.	µg/ml) after 24h in rat bone marrow (Mitotic Index (MI)			
Fontana et al. 2001	Increase micronuclei formation in exfoliated urothelial cells	Not Assessed	Positive	Not Assessed	Occupational exposure of mineral jelly (sodium nitrite N-phenyl-1-naphthylamine)
Ferrand et al. 2000	No significant changes	Not Assessed	Negative	Not Assessed	The chemical reaction products did not exhibit genotoxicity across all concentrations tested, as the repair ratios were close to 1
Jung et al. 1992	No significant changes	Not Assessed	Negative	Not Assessed	-
Luca et al. 1987	Increase micronuclei formation in bone marrow from mice exposed to sodium nitrite at concentrations of 1.72, 5.18, and 15.55.<br><br>Increase CA in bone marrow from rats and mice at all concentrations tested.<br><br>Increase CA in bone marrow from rabbits exposed to sodium	Not Assessed	Positive	Not Assessed	-

Authors	Genotoxicity assay	Cytotoxicity assay	Genotoxicity Outcome	Cytotoxicity Outcome	Observation
	nitrite at concentrations of 1.72, and 5.18.<br><br>Increase CA in BS-C-1 and HeLa cell lines at all concentrations tested.				

MN = micronucleus; CA = Chromosome Aberrations; SCE = Sister Chromatid Exchange
